# Supplementary figures and images for: Models of SIV rebound after treatment interruption that involve multiple reactivation events
Source: PLoS Comput Biol. 2020 Oct 1;16(10):e1008241. doi: 10.1371/journal.pcbi.1008241 (PMC7529301; doi:10.1371/journal.pcbi.1008241)

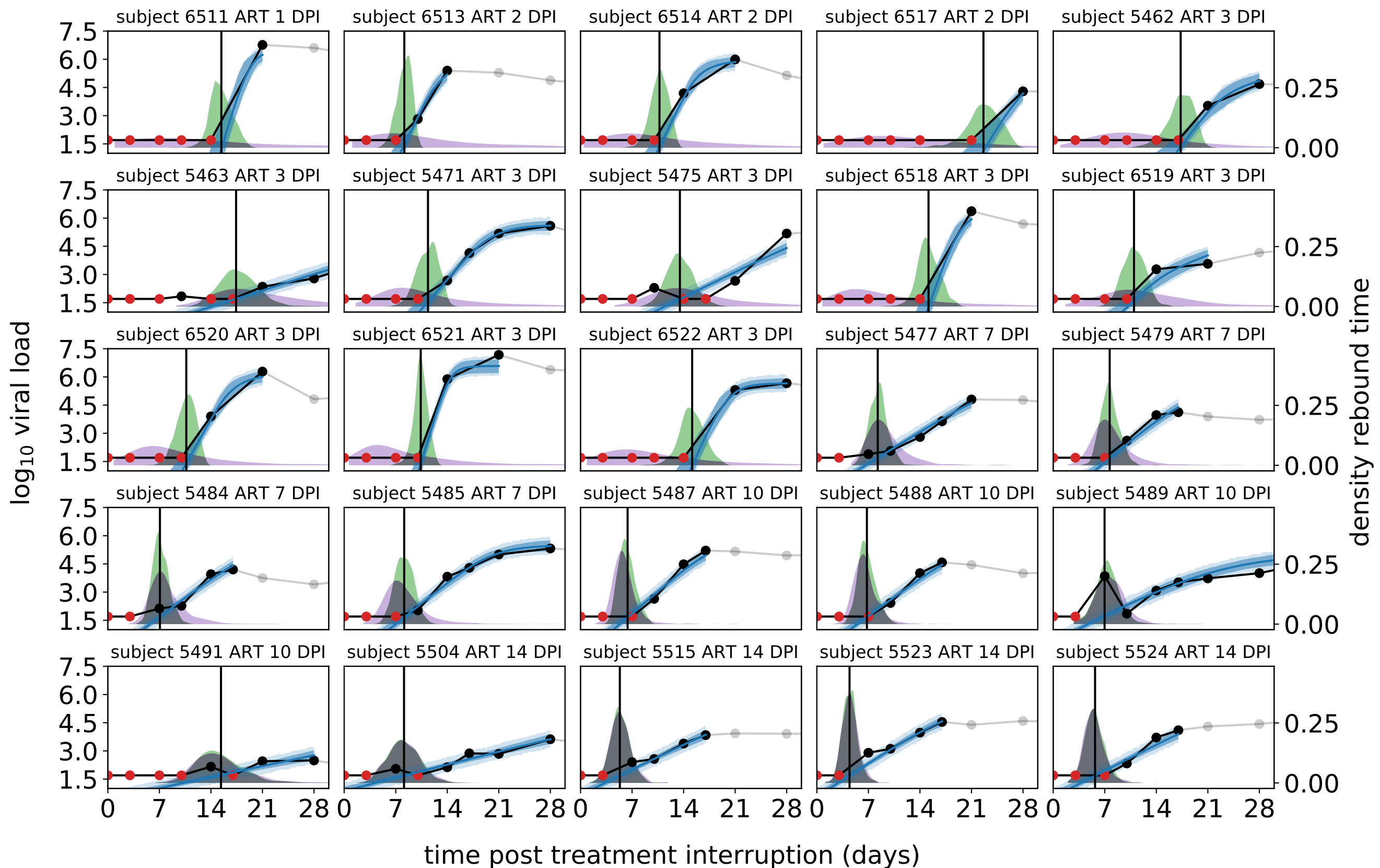

Supplement: S1 Fig — The panels (DPI: days post infection) show the VL data (black dots connected by black lines, with red dots for left-censored observations; the grey dots are ignored) taken from all 25 macaques for whom rebound was observed, and the stochastic multiple-reactivation model prediction (blue lines: posterior mean; dark blue band: 50% credible interval (CrI), light blue band: 50% posterior predictive interval). The estimated time-to-rebound (τ) is given by the vertical black line. The density plots in the background indicate the posterior predictive distribution of τ. The green distributions are conditioned on the estimated time of the initial recrudescence event, the purple distributions are unconditional. (PDF) [file pcbi.1008241.s001.pdf]

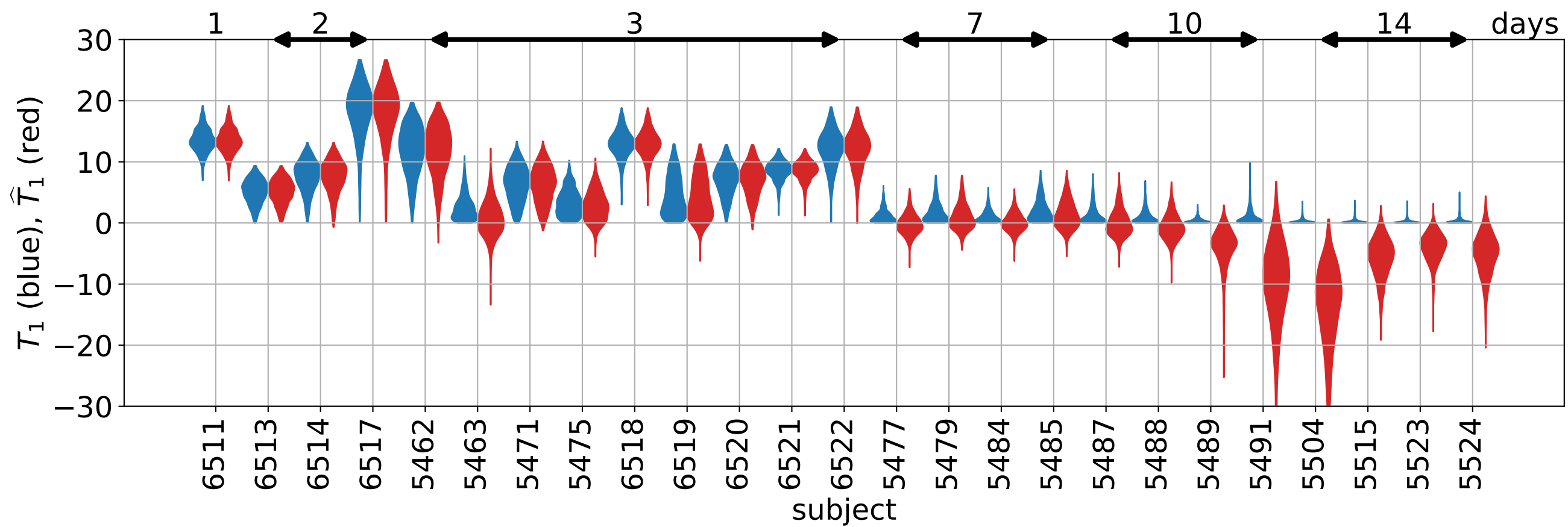

Supplement: S2 Fig — Marginal densities of T1 (blue) and the extrapolated T^1 (red) for each macaque are estimated with our multiple-reactivation model. The numbers on top indicate the time of ART initiation. (PDF) [file pcbi.1008241.s002.pdf]

**A**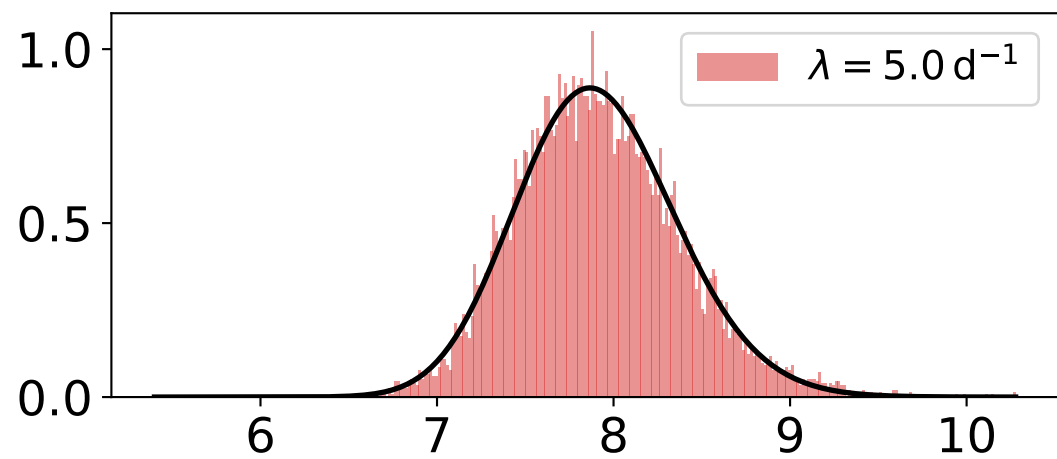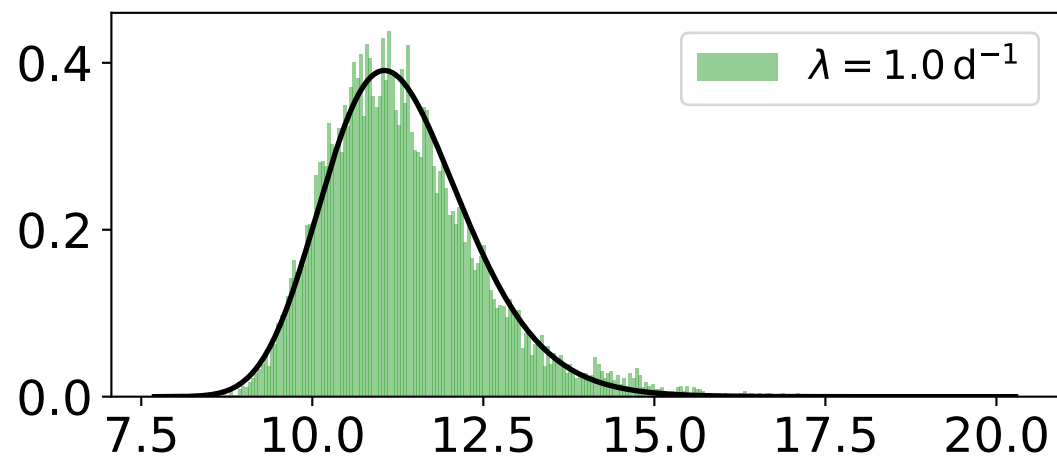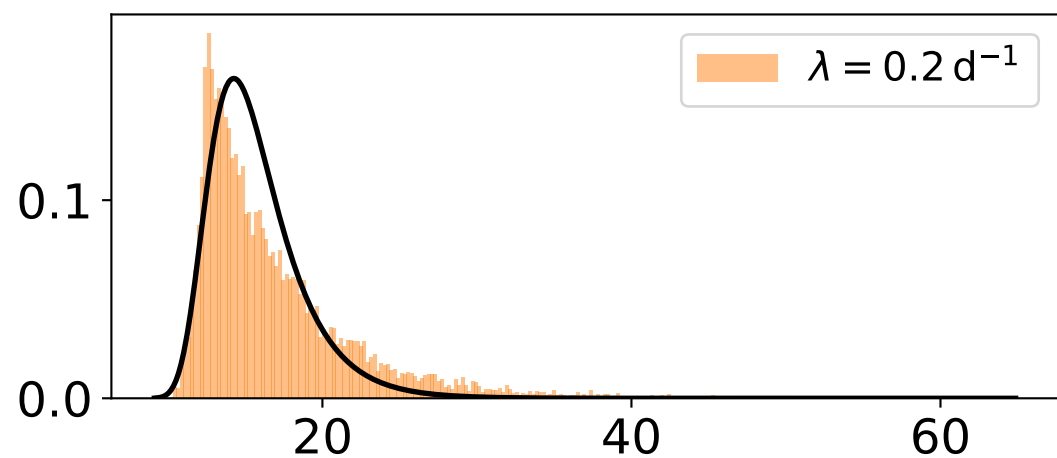**B**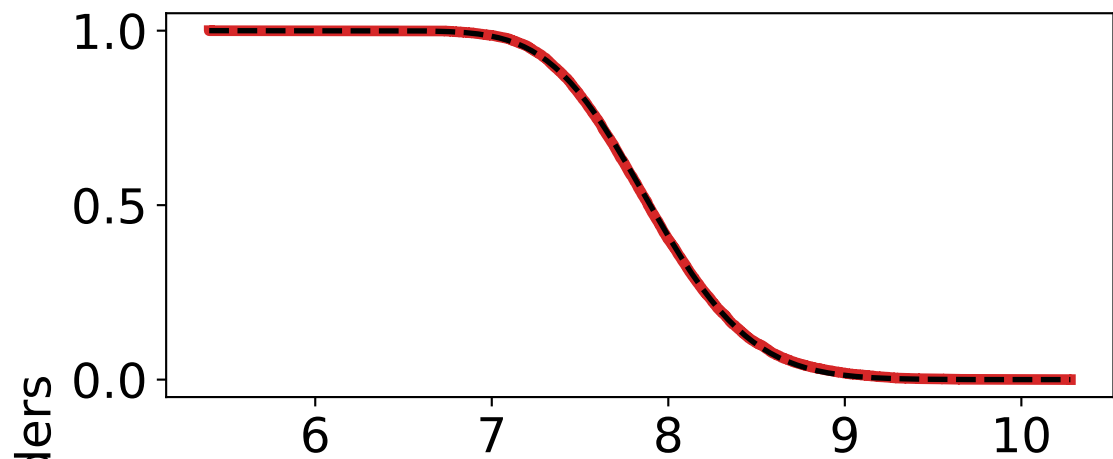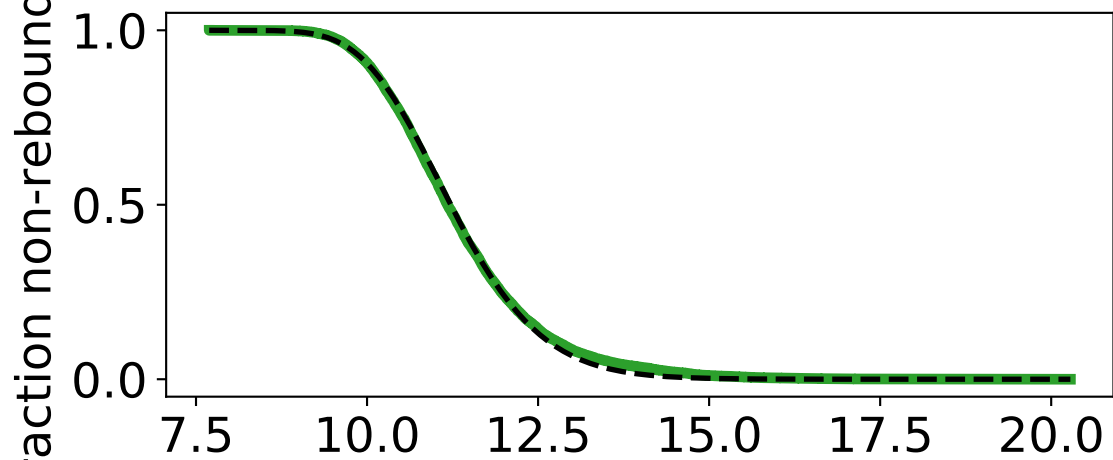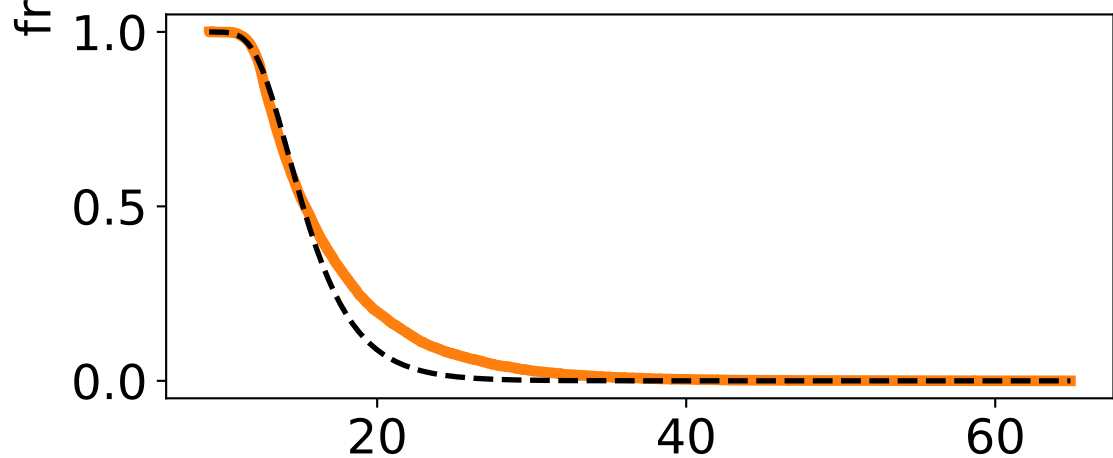

time to detection (days)

Supplement: S3 Fig — In this case, the law of Vt is approximated with a Gamma distribution with mean κ1(t) and variance κ2(t). The simulated empirical distributions are shown in color, and our approximation is shown in black. The predicted PDF (A) is calculated with numerical differentiation. (B) The survival function (i.e. the fraction of subjects S(t) that do not have a detectable VL at time t) is defined by Eq S5 in S1 Text. For the top, middle, and bottom panels different values of λ are used (λ = 5 d−1, 1 d−1, and 0.2 d−1 respectively). Notice the different time scale on the horizontal axes. For the remaining parameters, we used the values: g = 0.5 d−1, v0 = 0.1 copies mL−1, LoD ℓ = 50 copies mL−1. (PDF) [file pcbi.1008241.s003.pdf]

**A**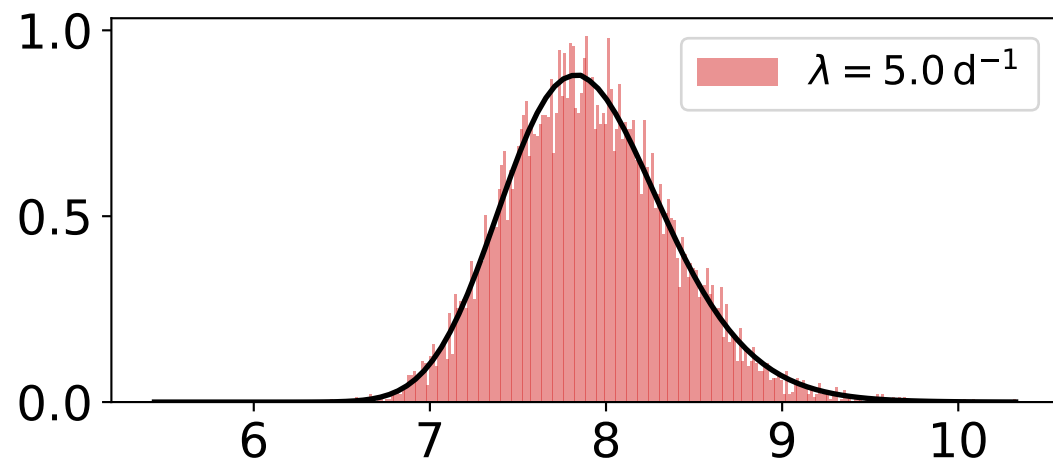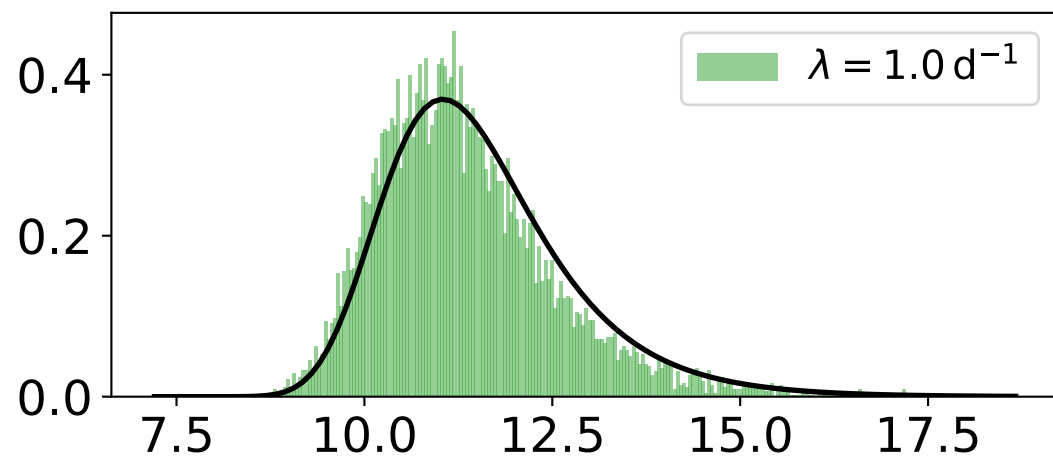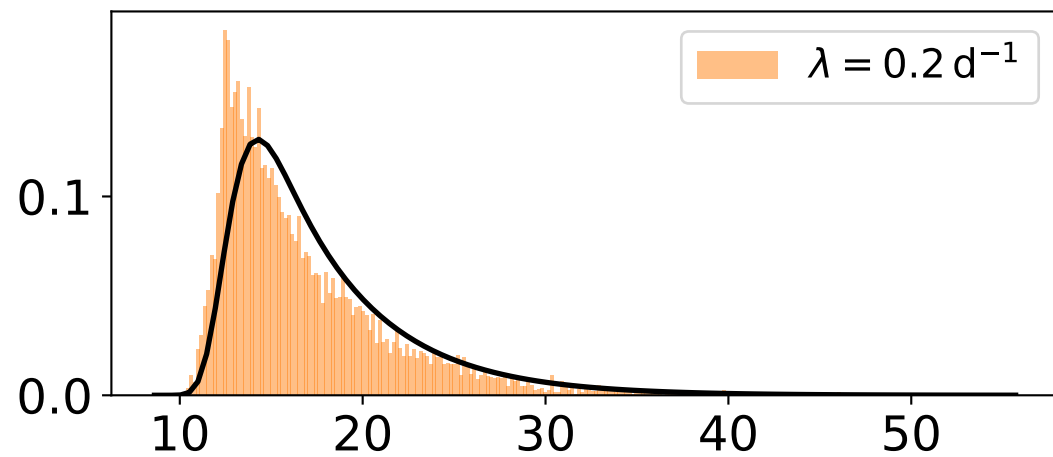**B**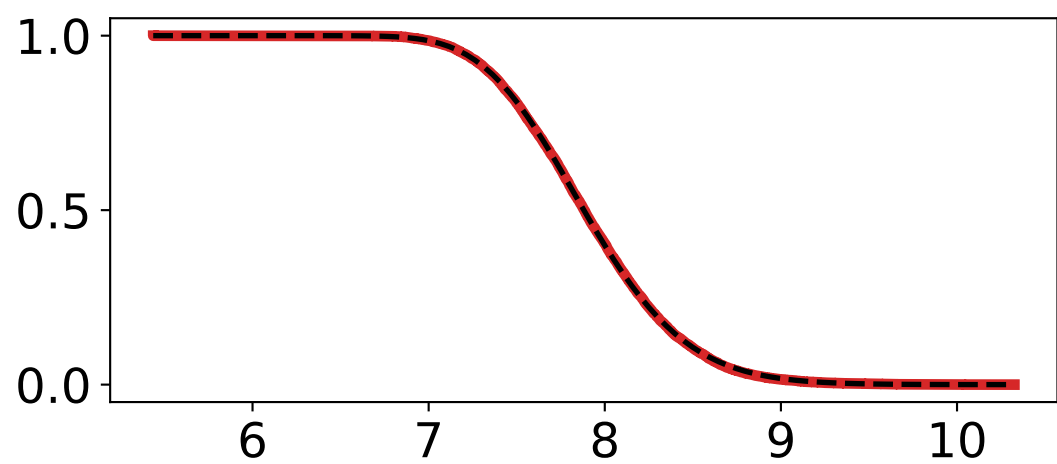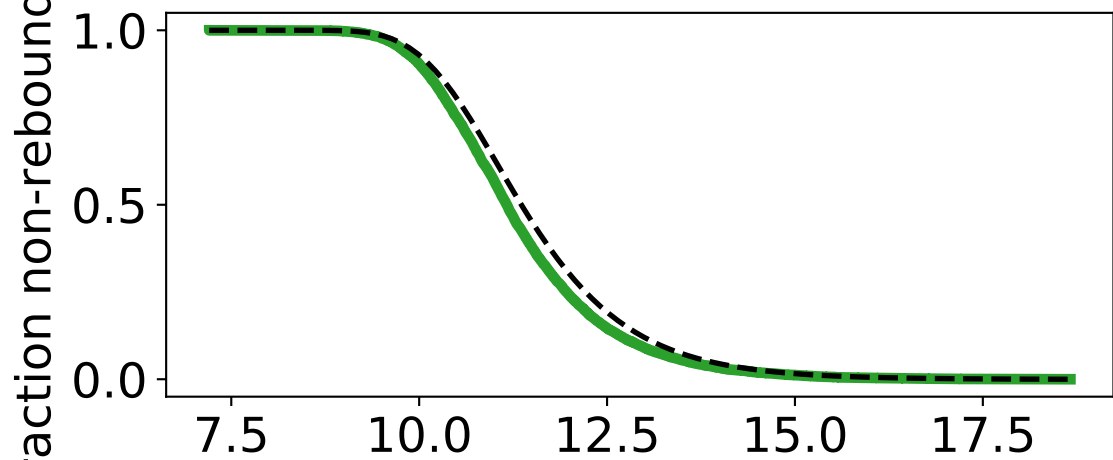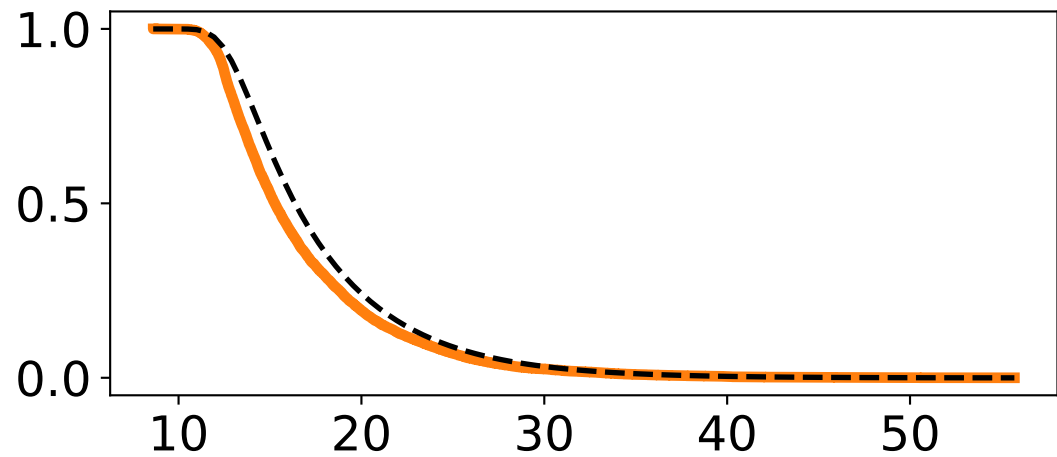

time to detection (days)

Supplement: S4 Fig — In this case, the master equation is approximated using the WKB ansatz. The simulated empirical distributions are shown in color, and our approximation is shown in black. (A) The probability density function (PDF; defined by Eq S11 and Eq S10 in S1 Text). (B) The survival function (i.e. the fraction of subjects S(t) that do not have a detectable VL at time t) is calculated with numerical integration. For the top, middle, and bottom panels different values of λ are used (λ = 5 d−1, 1 d−1, and 0.2 d−1 respectively). Notice the different time scale on the horizontal axes. For the remaining parameters, we used the values: g = 0.5 d−1, v0 = 0.1 copies mL−1, LoD ℓ = 50 copies mL−1. (PDF) [file pcbi.1008241.s004.pdf]

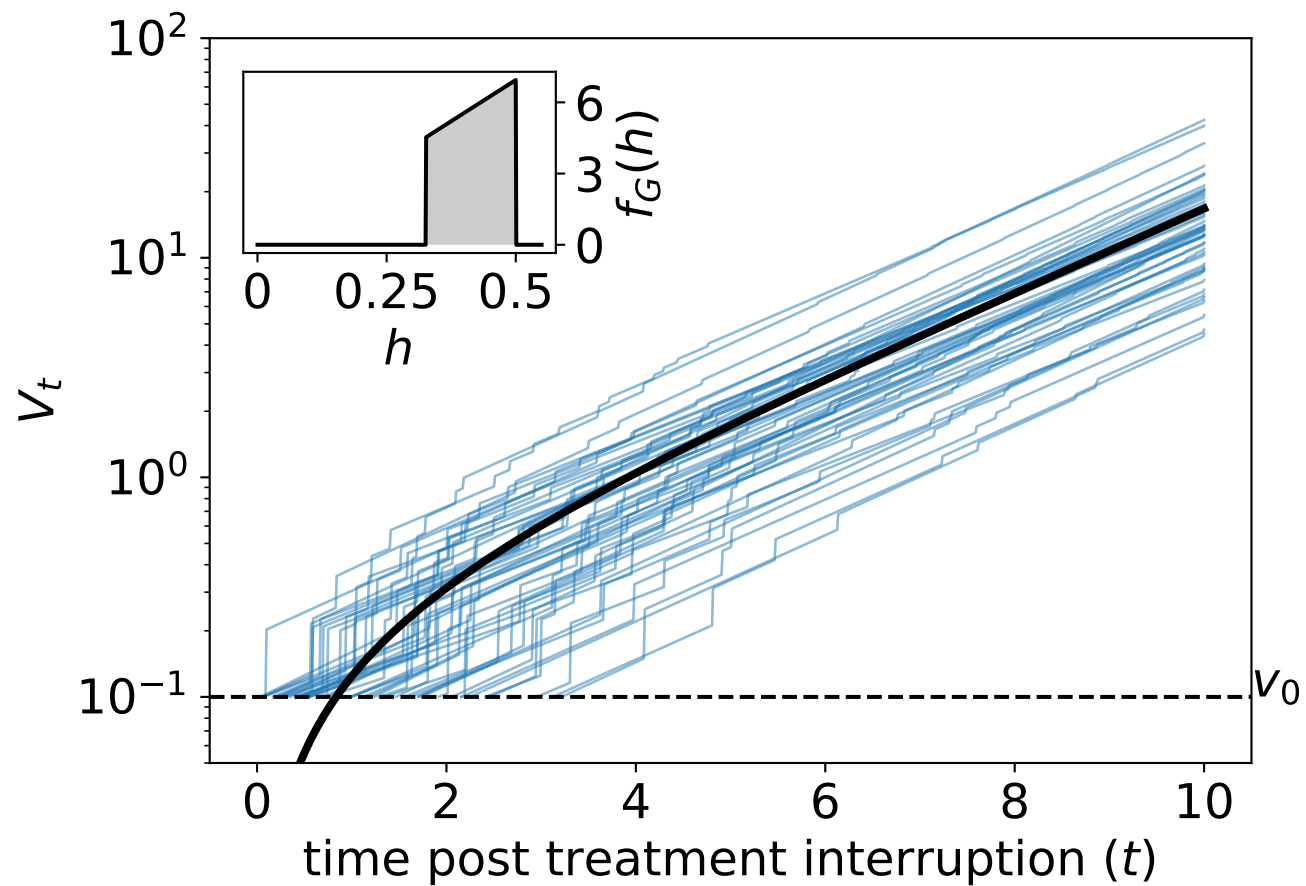

Supplement: S5 Fig — The black curve shows the expected value E[Vt]=κ1 (Eq S15). The inset shows the probability density function of the random growth rate Gi. The used parameter values are g = 0.5 d−1, σG = 0.05 d−1 (corresponding to u ≈ 0.175), v0 = 0.1 copies mL−1, and λ = 1 d−1. (PDF) [file pcbi.1008241.s005.pdf]

A

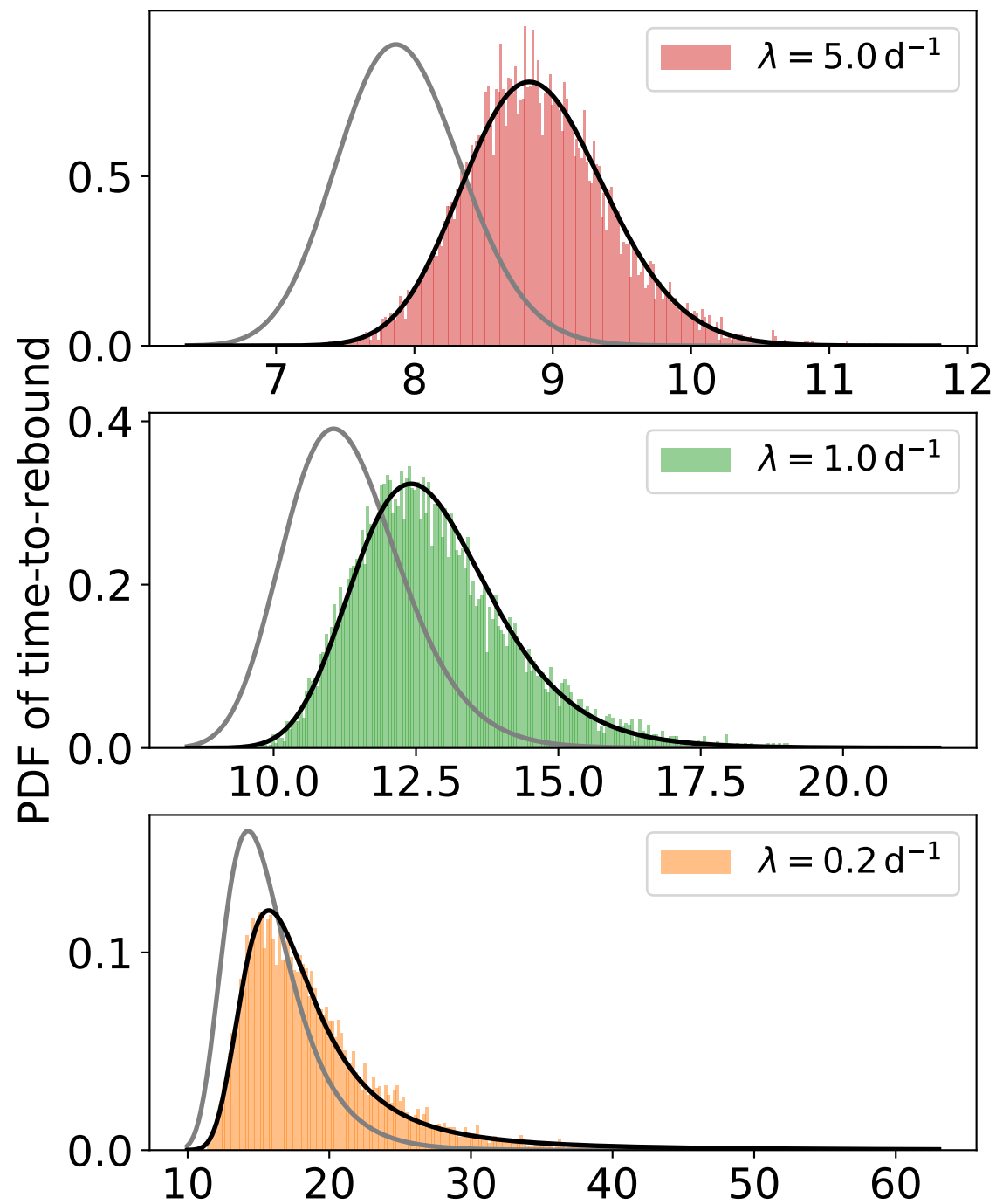

B

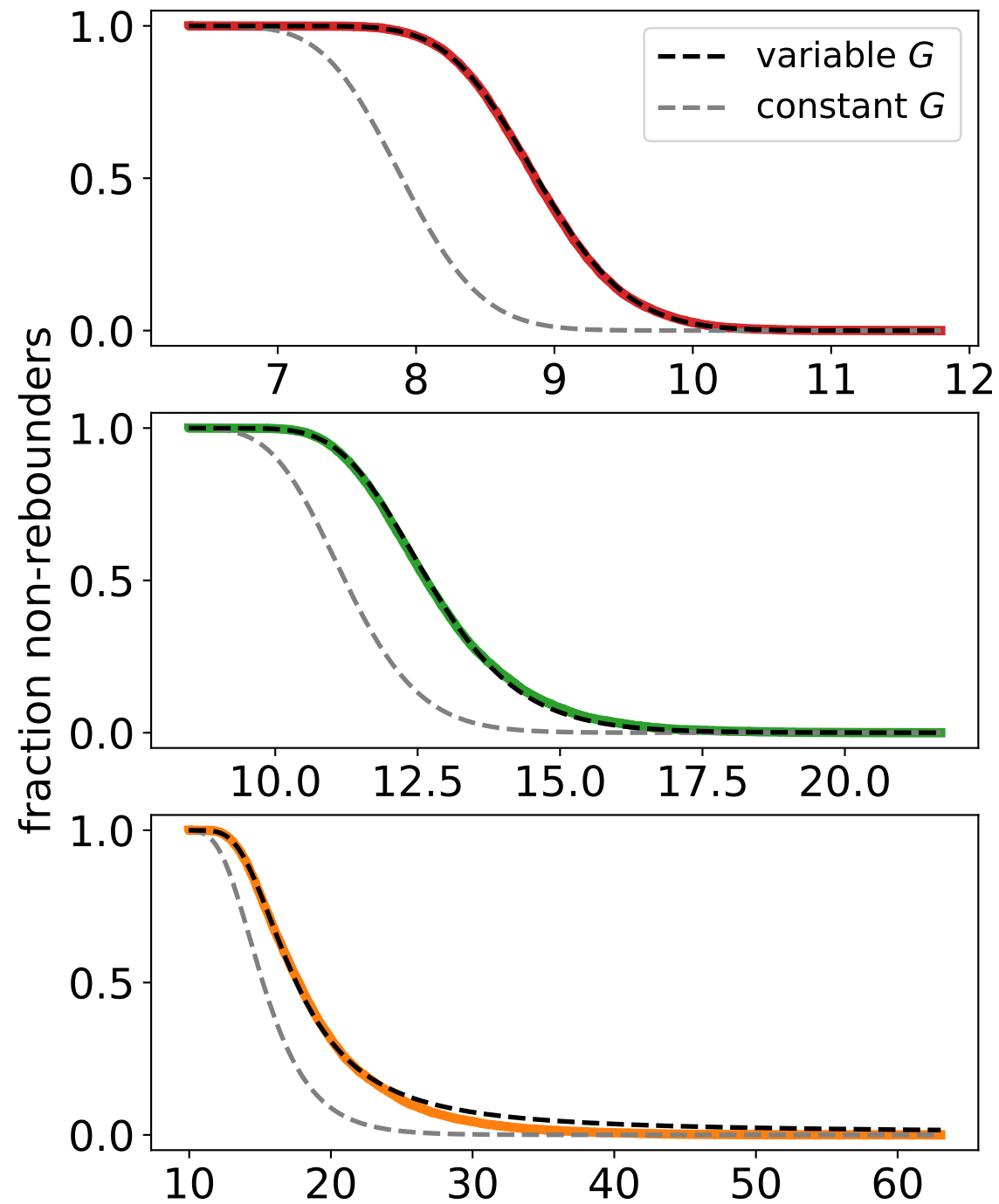

time to detection (days)

Supplement: S6 Fig — This model allows for variation in the exponential growth rate. The law of Vt is approximated with a Gamma distribution with mean κ1 (Eq S15 in S1 Text) and variance κ2 (Eq S16). The simulated empirical distributions are shown in color, and our approximation is shown in black. The predicted PDF (A) is calculated with numerical differentiation. (B) The survival function (i.e. the fraction of subjects S(t) that do not have a detectable VL at time t) is defined as S(t) = γ(k, ℓ/η) with γ the regularized incomplete Gamma function with parameters η = κ2/κ1 and k=κ12/κ2. For the top, middle, and bottom panels different values of λ are used (λ = 5 d−1, 1 d−1, and 0.2 d−1 respectively). Notice the different time scale on the horizontal axes. For the remaining parameters, we used the values: g = 0.5 d−1, σG = 0.05 d−1 (corresponding to u ≈ 0.175), v0 = 0.1 copies mL−1, LoD ℓ = 50 copies mL−1. The gray curves correspond to the approximate rebound time distribution with a constant growth rate (G ≡ g) and are identical to the black curves in S3 Fig. (PDF) [file pcbi.1008241.s006.pdf]
