# Supplementary material for: Models of SIV rebound after treatment interruption that involve multiple reactivation events
Source: PLoS Comput Biol. 2020 Oct 1;16(10):e1008241. doi: 10.1371/journal.pcbi.1008241 (PMC7529301; doi:10.1371/journal.pcbi.1008241)
Supplement: S1 Text — Here we derive the diffusion approximation of Vt by applying the Kramers-Moyal expansion to the master equation of the stochastic process Vt. Further, we explore two other approximations of Vt. First, we substitute the Gaussian distribution of Vt at time t with a Gamma distribution. Second, we replace the Kramers-Moyal expansion with the Wentzel–Kramers–Brillouin ansatz. Finally, we consider a generalization of the stochastic multiple-reactivation model that takes into account within-host variation in the exponential growth rate. We first derive the CGF, and then use the Gamma-distribution method to again derive an approximate rebound-time distribution for this generalized model. (PDF) [file pcbi.1008241.s007.pdf]

Supporting Text — Models of SIV rebound after treatment interruption that involve multiple reactivation events

Christiaan H. van Dorp[1,*], Jessica M. Conway[2], Dan H. Barouch[3,4], James B. Whitney[3,4], Alan S. Perelson[1]

1 Theoretical Biology and Biophysics (T-6), Los Alamos National Laboratory, Los Alamos, New Mexico, United States of America
2 Department of Mathematics and Center for Infectious Disease Dynamics, Pennsylvania State University, University Park, Pennsylvania, United States of America
3 Center for Virology and Vaccine Research, Beth Israel Deaconess Medical Center, Harvard Medical School, Boston, Massachusetts, United States of America
4 Ragon Institute of MGH, MIT, and Harvard, Cambridge, Massachusetts, United States of America

* chvandorp@gmail.com

Theoretical justification for the Gaussian approximation of V_t

In the main text, we derived that the stochastic process V_t with initial condition $V_0 = 0$ has mean $\kappa_1(t) = \lambda v_0 \frac{1}{g}(e^{gt} - 1)$ and variance $\kappa_2(t) = \lambda v_0^2 \frac{1}{2g}(e^{2gt} - 1)$. We then assumed that we could approximate the law (distribution) of V_t with $\mathcal{N}(\kappa_1(t), \kappa_2(t))$, from which we derived a probability distribution of the rebound time (see Materials and methods). Here we will give some additional mathematical arguments to justify this approach. We will first construct a stochastic differential equation (SDE) for V_t with jumps given by a Poisson process with intensity λ. We then infer the master equation for the process V_t, and use the Kramers-Moyal expansion to derive a Fokker-Planck equation for an approximation of V_t. In the SDE for this approximation the Poisson process is replaced by a Brownian motion with drift λ and diffusion $\sqrt{\lambda}$. The SDE for the approximation of V_t can be solved explicitly, as it is the SDE for a transient Ornstein-Uhlenbeck (OU) process. For more details about these techniques, see Van Kampen [1] and Steele [2].

The stochastic process V_t is the solution of the SDE

$$\mathrm{d}V_t = gV_t\,\mathrm{d}t + v_0\,\mathrm{d}N_t \tag{S1}$$

where N_t is a Poisson process with intensity λ. Let $\rho(t, v)$ denote the distribution of V_t. This distribution has a singular component as $\mathbb{P}[V_t = 0 | V_0 = 0] = e^{-\lambda t} \neq 0$, i.e. the VL is identically zero before the first recrudescence event. To avoid this complication, we assume that $v \gg v_0$. First, we derive the master equation for ρ. If $V_{t+h} = v$, and no reactivation has occurred in the time interval $(t, t+h]$, then V_t must have been equal to ve^{-gh}. The probability that no reactivation happened within this time interval is $1 - \lambda h$. On the other hand, if, with probability λh, a single reactivation did happen at time $T \in (t, t+h]$, the viral load V_t was equal to $ve^{-gh} - v_0 e^{g(t-T)}$. Conditional on $N_{t+h} = N_t + 1$, the jump time $T \sim \mathrm{Uniform}(t, t+h)$. Taking into account that probability is conserved, we get

$$\rho(t+h, v) = \rho(t, ve^{-gh})e^{-gh}(1 - \lambda h) + \lambda h \int_0^h \rho(t, ve^{-gh} - v_0 e^{gs})e^{-gh}\frac{\mathrm{d}s}{h} + o(h)$$

Using the mean-value theorem for integrals, we get that for some $s^* \in (0, h)$

$$\frac{\rho(t+h,v)-\rho(t,v)}{h} = \frac{\rho(t,ve^{-gh})e^{-gh}-\rho(t,v)}{h} + \lambda e^{-gh}\big(\rho(t,ve^{-gh}-v_0e^{-gs^*})-\rho(t,ve^{-gh})\big)+\frac{o(h)}{h}$$

and by taking the limit $h \to 0$, we find the master equation

$$\frac{\partial}{\partial t}\rho(t,v) = -g\frac{\partial}{\partial v}\big[v\rho(t,v)\big] + \lambda\big(\rho(t,v-v_0)-\rho(t,v)\big) \tag{S2}$$

As v_0 is small compared to v, we can use the Kramers-Moyal expansion to approximate the master equation. We first write

$$\rho(t,v-v_0) = \rho(t,v) - \frac{\partial}{\partial v}\rho(t,v)v_0 + \frac{1}{2}\frac{\partial^2}{\partial v^2}\rho(t,v)v_0^2 + \mathcal{O}(v_0^3)$$

and plug this into the master equation. When we ignore terms of order $\mathcal{O}(v_0^3)$, this results in the Fokker-Planck equation

$$\frac{\partial}{\partial t}\rho(t,v) = -g\frac{\partial}{\partial v}\big[(v+\tfrac{\lambda v_0}{g})\rho(t,v)\big] + \frac{1}{2}\lambda v_0^2\frac{\partial^2}{\partial v^2}\rho(t,v)$$

Notice that this Fokker-Planck equation corresponds to the SDE

$$\mathrm{d}V_t = g\left(V_t + \tfrac{\lambda v_0}{g}\right)\mathrm{d}t + \sqrt{\lambda}v_0\,\mathrm{d}B_t \tag{S3}$$

where B_t is a standard Brownian motion. Hence by taking the Kramers-Moyal expansion, we have replaced the Poisson process in the initial SDE (Eq S1) with $\sqrt{\lambda}B_t$, and we have added a drift term $\lambda v_0 \mathrm{d}t$. Eq S3 is up to a sign the SDE for the recurrent OU process and can be solved in a similar fashion. Let $X_t = e^{-gt}\big(V_t + \frac{\lambda v_0}{g}\big)$, then $\mathrm{d}X_t = e^{-gt}\sqrt{\lambda}v_0\mathrm{d}B_t$, which means that $X_t = X_0 + \sqrt{\lambda}v_0\int_0^t e^{-gs}\mathrm{d}B_s$. Therefore, X_t is a Gaussian process with mean X_0 and variance

$$\int_0^t \big(\sqrt{\lambda}v_0e^{-gs}\big)^2\mathrm{d}s = \frac{\lambda v_0^2}{2g}(1-e^{-2gt})$$

Since $V_t = e^{gt}X_t - \frac{\lambda v_0}{g}$, we find that V_t is a Gaussian process with mean $\frac{\lambda v_0}{g}(e^{gt}-1)+V_0e^{gt} = \kappa_1(t)+V_0e^{gt}$ and variance $\frac{\lambda v_0^2}{2g}(e^{2gt}-1) = \kappa_2(t)$.

Alternatives to the diffusion approximation

Heuristically imposing a Gamma law

Above we have used the Kramers-Moyal expansion in the master equation for V_t to justify replacing V_t with a transient OU process. This led to the approximate law $V_t \sim \mathcal{N}(\kappa_1(t), \kappa_2(t))$. However, the third cumulant of the true process V_t is positive, and hence V_t is right-skewed, whereas the normal distribution is not. This suggests that we could improve the approximation of the rebound-time distribution by replacing $\mathcal{N}(\kappa_1(t), \kappa_2(t))$ with a right-skewed distribution. This approach is heuristic as it lacks theoretical justification.

As an example, we consider the Gamma distribution with density $v \mapsto v^{k-1}e^{-v/\eta}\eta^{-k}\Gamma(k)^{-1}$, where Γ denotes the Gamma function. In order to match the

first and second moments, we must have $\kappa_1 = k\eta$ and $\kappa_2 = k\eta^2$. We therefore get the following expressions for k and η

$$k = \frac{\kappa_1^2}{\kappa_2} = \frac{2\lambda}{g}\tanh\left(\tfrac{1}{2}gt\right)\,, \quad \eta = \frac{\kappa_2}{\kappa_1} = \frac{v_0}{2}\left(e^{gt}+1\right) \tag{S4}$$

Here we have used the elementary identity $e^{2gt} - 1 = (e^{gt} - 1)(e^{gt} + 1)$.

Write $\tilde{\kappa}_3 = 2k\eta^3$ for the third cumulant of the Gamma distribution. Using our expression for η, we find

$$\tilde{\kappa}_3 = 2\kappa_2\eta = \frac{\lambda v_0^3}{2g}(e^{2gt}-1)(e^{gt}+1) \neq \kappa_3$$

and therefore the third cumulants of V_t and the matched Gamma distribution do not coincide. However, the relative difference between the two cumulants κ_3 and $\tilde{\kappa}_3$ is bounded, as for all $t \geq 0$ we have $\frac{3}{2} \leq \frac{\tilde{\kappa}_3}{\kappa_3} < 2$. In order to see this, we notice that

$$\frac{\tilde{\kappa}_3}{\kappa_3} = \frac{3}{2}\frac{e^{2gt}+2e^{gt}+1}{e^{2gt}+e^{gt}+1} = \frac{3}{2}\left(1 + \frac{1}{e^{gt}+1+e^{-gt}}\right) \to \begin{cases} \frac{3}{2} & \text{as } t \to \infty \\ 2 & \text{as } t \to 0 \end{cases}$$

and that $e^{gt} + 1 + e^{-gt}$ is a non-decreasing function of t.

Following the same steps as with the Gaussian case, we get the following survival function $S(t)$ for the rebound time

$$S(t;\lambda,g,v_0,\ell) = \gamma\left(\tfrac{2\lambda}{g}\tanh(\tfrac{1}{2}gt), 2\ell v_0^{-1}(e^{gt}+1)^{-1}\right) \tag{S5}$$

where $\gamma(a,x) = \frac{1}{\Gamma(a)}\int_0^x e^{-s}s^{a-1}\mathrm{d}s$ denotes the regularized incomplete Gamma function.

To prove that S is a proper survival function, we have to show that S is a monotonically non-increasing function of t. The derivative of S is equal to

$$\frac{\mathrm{d}S}{\mathrm{d}t} = \frac{\partial\gamma}{\partial a}\frac{\mathrm{d}k}{\mathrm{d}t} + \frac{\partial\gamma}{\partial x}\frac{\mathrm{d}}{\mathrm{d}t}\ell\eta^{-1}$$

As η and k are monotonically increasing functions of t (see Eq S4), and γ is a monotonically non-decreasing function of x, we only have to verify that γ as a function of a is monotonically non-increasing. Using a change of variables $s = ux$, and splitting the Gamma function into the sum of two integrals, we get the following expression for the regularized incomplete Gamma function

$$\gamma(a,x) = \frac{I_1}{I_1+I_2} \quad \text{with} \quad I_1 \equiv \int_0^1 e^{-ux}u^{a-1}\,\mathrm{d}u \quad \text{and} \quad I_2 \equiv \int_1^\infty e^{-ux}u^{a-1}\,\mathrm{d}u$$

The integrand of I_1 is a monotonically non-increasing function of a, because the integration variable $u \in [0,1]$. Conversely, the integrand of I_2 is a monotonically non-decreasing function of a. Hence, γ monotonically decreases as a function of a. This shows that S is indeed monotonically non-increasing.

In S3 Fig, we compare the rebound-time distribution corresponding to survival function (Eq S5) with simulations, using three different recrudescence rates λ. Comparing Fig 2 and S3 Fig shows that the diffusion approximation and the Gamma-based approximation perform equally well for $\lambda = 5\,\mathrm{d}^{-1}$, and $1\,\mathrm{d}^{-1}$. When successful reactivation events are rare ($\lambda = 0.2\,\mathrm{d}^{-1}$), the approximation based on the Gamma law outperforms the diffusion approximation. However, the Gamma-based approximation is still unable to capture the exponential tail of the time-to-rebound distribution that is visible in the simulations with a small recrudescence rate.

The probability-density function of the time-to-rebound distribution is equal to $f(t) = -\frac{\mathrm{d}}{\mathrm{d}t} S(t)$. Using e.g. Mathematica [3], it is possible to obtain an expression for f in terms of a variety of special functions, which are not available in many other software packages. However, if the data of interest consists solely of interval- and right-censored rebound times and subsequent VL observations are not used to estimate the exact instance that the VL became observable, the density f is not required and the survival function S can be used directly (cf. [4]) to calculate the likelihood of the data.

The WKB approximation of the master equation.

In addition to the heuristic attempt to improve the approximation of the rebound-time distribution, we here explore a more advanced approach in which we replace the Kramers-Moyal expansion of the master equation (Eq S2) with the Wentzel-Kramers-Brillouin (WKB) *ansatz*. For details about this technique, we refer to e.g. Friedlin and Wentzell [5]. Assuming that v_0 is a small parameter, the WKB *ansatz* suggests that we write $\rho(t,v) \propto e^{-v_0^{-1}\mathcal{S}(t,v)}$ for some function $\mathcal{S}$ (not to be confused with the survival function S). When we substitute $\rho = Ce^{-v_0^{-1}\mathcal{S}}$ in the master equation, and divide everything by ρv_0^{-1}, we get

$$-\tfrac{\partial \mathcal{S}}{\partial t} = -gv_0 + gv\tfrac{\partial \mathcal{S}}{\partial v} + \lambda v_0 \left[e^{-v_0^{-1}(\mathcal{S}(t,v-v_0)-\mathcal{S}(t,v))} - 1\right] \tag{S6}$$

We now take a first-order Taylor expansion of $\mathcal{S}(t, v - v_0) = \mathcal{S}(t,v) - v_0 \frac{\partial \mathcal{S}}{\partial v}(t,v) + \mathcal{O}(v_0^2)$ around v and when we ignore terms of order $\mathcal{O}(v_0)$, we can write

$$\exp\left(-v_0^{-1}\left(\mathcal{S}(t,v-v_0) - \mathcal{S}(t,v)\right)\right) \approx \exp\left(\tfrac{\partial \mathcal{S}}{\partial v}\right)$$

Again ignoring terms of order $\mathcal{O}(v_0)$, Eq S6 simplifies to

$$-\tfrac{\partial \mathcal{S}}{\partial t} = gv\tfrac{\partial \mathcal{S}}{\partial v} + \lambda v_0 \left[\exp\left(\tfrac{\partial \mathcal{S}}{\partial v}\right) - 1\right] \tag{S7}$$

Here we have to assume that $\lambda^{-1} = \mathcal{O}(v_0)$ as $v_0 \to 0$, but below we will see that our results hold for small λ as well. Hence, we have replaced the master equation for V_t, which is both a functional and partial differential equation, with the first-order non-linear PDE in Eq S7, which can be solved with the method of characteristics. Eq S7 has the form of a Hamilton-Jacobi equation $-\frac{\partial \mathcal{S}}{\partial t} = \mathcal{H}\left(v, \frac{\partial \mathcal{S}}{\partial v}\right)$ with Hamiltonian $\mathcal{H}(v,p) = gvp + \lambda v_0(e^p - 1)$ and we find the canonical equations (see e.g. [6])

$$\begin{aligned} \tfrac{\mathrm{d}v}{\mathrm{d}t} &= \tfrac{\partial \mathcal{H}}{\partial p} = gv + \lambda v_0 e^p \\ \tfrac{\mathrm{d}p}{\mathrm{d}t} &= -\tfrac{\partial \mathcal{H}}{\partial v} = -gp \end{aligned}$$

which can be solved explicitly. First, we find that $p(t) = p_0 e^{-gt}$, and we get a first order ODE for v with time-dependent parameters and initial condition $v(0) = 0$. This ODE has solution

$$v(t;p_0) = \frac{\lambda v_0}{g p_0} e^{gt}\left(e^{p_0} - e^{p_0 e^{-gt}}\right) \tag{S8}$$

If we take the limit $p_0 \to 0$ in Eq S8, we get $v(t;0) = \frac{\lambda v_0}{g}(e^{gt} - 1)$, which is the trajectory of the expectation of V_t. A solution of the PDE in Eq S7 can now we derived by integrating the Lagrangian associated with $\mathcal{H}$ along the characteristic paths $v(t;p_0)$. This Lagrangian $\mathcal{L}$ is given by

$$\mathcal{L}(v,\dot{v}) = p\dot{v} - \mathcal{H}(v,p) = \lambda v_0 \left(e^{p_0 e^{-gt}}(p_0 e^{-gt} - 1) + 1\right)$$

Here we write $\dot{v}$ to denote the time-derivative of v. Hamilton's principal function is then given by the integral of the Lagrangian along a characteristic path $\{v(s;p_0) : s \in [0,t]\}$, i.e.

$$\begin{aligned}\mathcal{S}(t;p_0) &\equiv \int_0^t \mathcal{L}\big(v(s;p_0), \dot{v}(s;p_0)\big)\,\mathrm{d}s \\ &= \lambda v_0 t + \frac{\lambda v_0}{g}\left(e^{p_0} - e^{p_0 e^{-gt}} - \mathrm{Ei}(p_0) + \mathrm{Ei}(p_0 e^{-gt})\right)\end{aligned}$$

where $\mathrm{Ei}(x) \equiv \int_{-\infty}^x e^s s^{-1}\mathrm{d}s$ denotes the exponential integral. In order to find a solution $\mathcal{S}(t,x)$ of Eq S7, we have to find a $p_0 = p_0(t,x)$ such that $v(t, p_0(t,x)) = x$. Then $\mathcal{S}(t,x) = \mathcal{S}(t; p_0(t,x))$.

It turns out that as an approximation of the rebound time, we can simply take

$$f(t;\lambda,g,v_0,\ell) \propto \exp\big(-v_0^{-1}\mathcal{S}(t,\ell)\big) \tag{S9}$$

which can be made precise using the theory of large deviations [5]. In S4 Fig, we have compared the rebound-time distribution derived using the WKB approximation with simulated rebound times. Comparing this with Fig 2 and S3 Fig, we find a significant improvement in the accuracy when λ is small. Hence, the WKB approximation is much better at describing the exponential tail of the rebound-time distribution that is due to the exponential waiting time of the first successful reactivation.

However, in order to apply this method, we have to solve the equation $v(t;p_0) = \ell$ for p_0, and find a constant that normalizes f in Eq S9. Both of these problems have to be solved numerically, which makes the method difficult to implement in a parameter-inference framework. We can somewhat simplify the equations by taking two limits. First, the process V_t is nearly deterministic above the detection limit. This is reflected by the fact that the Lagrangian vanishes as t becomes large. So instead of integrating the Lagrangian from 0 to t (assuming that $v(t;p_0) = \ell$), we might as well integrate from 0 to ∞, as the contribution from the interval (t,∞) is negligible. In this case, Hamilton's principal function is given by

$$\mathcal{S}(p_0) = \frac{\lambda v_0}{g}\left(e^{p_0} - 1 + \mathrm{Ein}(-p_0)\right) \tag{S10}$$

where the function $\mathrm{Ein}(x) \equiv \int_0^x (1-e^{-s})s^{-1}\mathrm{d}s$ can be expressed in terms of other exponential integrals.

Second, we can make use of an asymptotic symmetry which is again due to the near determinism as V_t becomes large. Let $L > \ell$ be some VL level much larger than the LoD ℓ. As V_t grows exponentially, it takes about $\frac{1}{g}\log(L/\ell)$ days to reach level L starting at LoD ℓ. This means that the parameter p_0 that solves $\ell = v(t;p_0)$ must be nearly identical to the solution of $L = v\big(t + \frac{1}{g}\log(L/\ell); p_0\big)$. The latter equation can be re-arranged as

$$L = \frac{\lambda v_0}{g p_0} e^{gt}\frac{L}{\ell}\left(e^{p_0} - e^{p_0 e^{-gt}\frac{\ell}{L}}\right)$$

After dividing by both sides of the equation by L, we can take the limit $L \to \infty$, and we get the following equation for p_0

$$p_0 = \frac{\lambda v_0}{g\ell} e^{gt}(e^{p_0} - 1) \tag{S11}$$

This equation can be solved in terms of the Lambert W function. We used Eq S11 together with Eq S10 to plot the curves in S4 Fig. Despite these simplifications, using this method for inference would still be difficult due to the unknown normalizing constant in Eq S9.

Incorporating within-host variation in the exponential growth rate

In the models described above, we have assumed that the exponential growth rate g is constant within a host. Here we generalize the model so that we can incorporate variation in this growth rate. We assume again that recrudescence happens according to a Poisson process at constant rate λ. At each recrudescence time T_i, a realization of the random variable G_i is sampled, which determines the growth rate of the i-th successfully reactivating clone. For mathematical tractability, we have to assume that the G_i are independent from each other and from T_i and identically distributed. In reality, this is not necessarily true, as the growth rate is related to viral fitness and clones with a higher fitness are more likely to reactivate successfully. The viral load process V_t at time t after treatment interruption is now given by

$$V_t = v_0 \sum_{i=1}^{\infty} \mathbb{1}_{[T_i,\infty)}(t) e^{G_i(t-T_i)} \tag{S12}$$

Example realizations of this process are shown in S5 Fig. As before, we can derive the cumulant-generating function $K(\theta) = \log \mathbb{E}[\exp(\theta V_t)]$, but now we have to take into account that the growth rates G_i are random variables. We first condition on $N_t = n$ as before, and get

$$\mathbb{E}[\exp(\theta V_t)] = \prod_{i=1}^{n} \mathbb{E}\left[\exp\left(\theta v_0 e^{G_i(t-T_i)}\right)\right] = \left(\frac{1}{t}\int_0^t \mathbb{E}[\exp(\theta v_0 e^{Gs})]\mathrm{d}s\right)^n$$

Here the expectations are conditional on $N_t = n$ and G is identically distributed as any one of the G_i. Now, we sum over all possible n and take the logarithm to get

$$K(\theta) = \log \mathbb{E}[\exp(\theta V_t)] = \lambda \int_0^t \mathbb{E}[\exp(\theta v_0 e^{Gs})]\mathrm{d}s - \lambda t \tag{S13}$$

Notice that we now require that the moment generating function of $v_0 \exp(Gs)$ exists, which is true when e.g. G is bounded, but not the case for arbitrary distributions of G. Now we can again extract the first and second cumulant by evaluating the first and second derivative of $K(\theta)$ at $\theta = 0$:

$$\kappa_1 = \lambda v_0 \int_0^t \mathbb{E}[\exp(Gs)]\mathrm{d}s\,, \quad \kappa_2 = \lambda v_0^2 \int_0^t \mathbb{E}[\exp(2Gs)]\mathrm{d}s \tag{S14}$$

Here we require that the distribution of G is well-behaved enough such that we can interchange differentiation and taking the expectation. Again, this is true when we make the biologically plausible assumption that G is bounded.

To proceed from here, we have to choose a probability distribution for the growth rate G. As an example, we choose a convenient distribution that results in simple elementary expressions for κ_1 and κ_2. We hypothesize that clones with a higher growth rate (fitness) constitute a larger part of the reservoir, for instance because they could have been more common during acute infection. The most common clone in the reservoir has the growth rate g, and all other clones are less fit and have growth rates h in the interval $[g-u, g]$, with likelihood proportional to h. Hence, the distribution of G is given by the PDF $f_G(h) = \frac{h}{u(g-u/2)}\mathbb{1}_{[g-u,g]}(h)$ (see the inset of S5 Fig). The variance of G is equal to $\sigma_G^2 \equiv \frac{u^2(g^2-gu+u^2/6)}{12(g-u/2)^2}$ and can be adjusted by choosing the width u of the domain of G.

With this choice for the distribution of G, we get

$$\begin{aligned}\kappa_1 &= \frac{\lambda v_0}{u(g-u/2)} \int_0^t \int_{g-u}^g e^{hs} h \,\mathrm{d}h \,\mathrm{d}s = \frac{\lambda v_0}{u(g-u/2)} \int_{g-u}^g (e^{ht}-1) \,\mathrm{d}h \\ &= \frac{\lambda v_0}{g-u/2} \left(e^{gt} \frac{1-e^{-ut}}{ut} - 1 \right)\end{aligned} \tag{S15}$$

Notice that the factor h in the first integrand ensures that we get an elementary expression for κ_1. Similarly, we get

$$\kappa_2 = \frac{v_0^2 \lambda}{2g-u} \left(e^{2gt} \frac{1-e^{-2ut}}{2ut} - 1 \right) \tag{S16}$$

Now that we have expressions for the mean (κ_1) and variance (κ_2) of V_t, we can again construct an approximate probability density function of the rebound time τ by approximating the distribution of V_t with a convenient probability distribution that has the same mean and variance. In this case, we can not take the normal distribution, as the z-score $\frac{\ell-\kappa_1}{\sqrt{\kappa_2}}$ of the LoD ℓ is not a monotone, decreasing function of t. However, we can still use the our heuristic Gamma law instead of a normal distribution, with parameters $k = \frac{\kappa_1^2}{\kappa_2}$ and $\eta = \frac{\kappa_2}{\kappa_1}$. Although we could not mathematically prove that this method resulted in a well-defined survival function and PDF, we verified numerically that for biologically plausible parameters and time windows the survival function is monotonically non-increasing, and that the PDF is non-negative. The resulting PDF and survival function are compared to simulated rebound times in S6 Fig. In the same figure, we have repeated the approximate rebound time distributions derived from the model with a fixed growth rate g (S6 Fig, gray curves). This shows clearly that viral rebound is delayed in the case of a variable growth rate. This is to be expected, because the first clones that reactivate might have a smaller exponential growth rate (between $g-u$ and g), and take longer to reach the limit of detection. Eventually, a clone with a growth rate close to g will successfully reactivate.

References

1. van Kampen NG. Stochastic processes in physics and chemistry. 3rd ed. Amsterdam: Elsevier; 2007.

2. Steele JM. Stochastic calculus and financial applications. New York: Springer; 2001.

3. Wolfram Research, Inc. Mathematica, version 11.2; 2017.

4. Conway JM, Perelson AS, Li JZ. Predictions of time to HIV viral rebound following ART suspension that incorporate personal biomarkers. PLoS Comput Biol. 2019;15(7):1–26. doi:10.1371/journal.pcbi.1007229.

5. Freidlin MI, Wentzell AD. Random perturbations of dynamical systems. 3rd ed. Grundlehren der mathematischen Wissenschaften. Berlin, Heidelberg: Springer; 2012.

6. Gelfand IM, Fomin SV. Calculus of variations. Dover Publications; 2012.
