# Supplementary material for: Models of SIV rebound after treatment interruption that involve multiple reactivation events
Source: PLoS Comput Biol. 2020 Oct 1;16(10):e1008241. doi: 10.1371/journal.pcbi.1008241 (PMC7529301; doi:10.1371/journal.pcbi.1008241)
Supplement: S1 Table — Parameter estimates from the fully stochastic multi-reactivation model (“rebound” columns). and the acute infection (“acute” columns). The point estimates correspond to the mode of the marginal posterior distributions. (PDF) [file pcbi.1008241.s008.pdf]

| | | *rebound* | | | | *acute* | |
|---|---|---|---|---|---|---|---|
| *population-level parameters* | | | | | | | |
| symbol | unit | mode | 95% CrI | | | mode | 95% CrI |
| $\alpha_g$ | $\log \mathrm{d}^{-1}$ | $-0.48$ | $[-0.95, -0.18]$ | | | | |
| $\alpha_\lambda$ | $\log \mathrm{d}^{-1}$ | 1.47 | $[0.73, 2.10]$ | | | | |
| $\mu_g$ | $\log \mathrm{d}^{-1}$ | $-0.24$ | $[-0.52, 0.52]$ | | | 0.67 | $[0.58, 0.79]$ |
| $\mu_\lambda$ | $\log \mathrm{d}^{-1}$ | $-0.93$ | $[-1.65, -0.01]$ | | | | |
| $\sigma_g$ | $\log \mathrm{d}^{-1}$ | 0.63 | $[0.44, 1.33]$ | | | 0.05 | $[0.01, 0.27]$ |
| $\sigma_\lambda$ | $\log \mathrm{d}^{-1}$ | 0.12 | $[0.02, 0.95]$ | | | | |
| $v_0$ | copies $\mathrm{mL}^{-1}$ | 0.36 | $[0.11, 1.89]$ | | | | |
| $\sigma$ | log copies $\mathrm{mL}^{-1}$ | 1.28 | $[1.05, 1.63]$ | | | 0.80 | $[0.51, 1.12]$ |
| *individual-level parameters* | | | | | | | |
| subject | start ART (d) | $g$ ($\mathrm{d}^{-1}$) | 95% CrI | $\lambda$ ($\mathrm{d}^{-1}$) | 95% CrI | $g$ ($\mathrm{d}^{-1}$) | 95% CrI |
| 6511 | 1 | 2.21 | $[1.24, 7.43]$ | 0.07 | $[0.03, 0.19]$ | | |
| 6513 | 2 | 1.37 | $[0.85, 4.48]$ | 0.10 | $[0.04, 0.30]$ | | |
| 6514 | . | 1.30 | $[0.77, 4.62]$ | 0.10 | $[0.04, 0.28]$ | | |
| 6517 | . | 1.01 | $[0.44, 4.60]$ | 0.08 | $[0.04, 0.20]$ | | |
| 5462 | 3 | 0.70 | $[0.42, 3.65]$ | 0.13 | $[0.06, 0.31]$ | | |
| 5463 | . | 0.27 | $[0.20, 0.43]$ | 0.14 | $[0.07, 0.56]$ | | |
| 5471 | . | 0.82 | $[0.56, 1.99]$ | 0.13 | $[0.06, 0.35]$ | | |
| 5475 | . | 0.42 | $[0.32, 0.63]$ | 0.14 | $[0.06, 0.50]$ | | |
| 6518 | . | 1.86 | $[1.09, 5.53]$ | 0.12 | $[0.05, 0.29]$ | | |
| 6519 | . | 0.56 | $[0.38, 2.88]$ | 0.14 | $[0.06, 0.42]$ | 1.95 | $[1.56, 2.59]$ |
| 6520 | . | 1.19 | $[0.75, 4.05]$ | 0.13 | $[0.06, 0.38]$ | | |
| 6521 | . | 2.36 | $[1.32, 5.82]$ | 0.13 | $[0.06, 0.32]$ | | |
| 6522 | . | 1.34 | $[0.76, 5.33]$ | 0.13 | $[0.06, 0.29]$ | | |
| 5477 | 7 | 0.56 | $[0.41, 0.74]$ | 0.45 | $[0.21, 1.96]$ | 1.98 | $[1.73, 2.65]$ |
| 5479 | . | 0.67 | $[0.50, 1.31]$ | 0.42 | $[0.19, 1.78]$ | 1.97 | $[1.64, 2.34]$ |
| 5484 | . | 0.65 | $[0.47, 0.94]$ | 0.46 | $[0.21, 2.45]$ | 2.02 | $[1.81, 2.97]$ |
| 5485 | . | 0.65 | $[0.49, 1.18]$ | 0.42 | $[0.18, 1.62]$ | 1.93 | $[1.57, 2.22]$ |
| 5487 | 10 | 0.71 | $[0.54, 1.20]$ | 0.92 | $[0.35, 4.96]$ | 1.90 | $[1.60, 2.21]$ |
| 5488 | . | 0.60 | $[0.46, 1.00]$ | 0.93 | $[0.34, 5.94]$ | 1.96 | $[1.71, 2.50]$ |
| 5489 | . | 0.39 | $[0.26, 1.05]$ | 1.38 | $[0.41, 12.64]$ | 1.91 | $[1.55, 2.25]$ |
| 5491 | . | 0.19 | $[0.09, 0.31]$ | 0.88 | $[0.36, 5.01]$ | 1.93 | $[1.62, 2.36]$ |
| 5504 | 14 | 0.23 | $[0.13, 0.35]$ | 3.04 | $[0.76, 28.52]$ | 2.00 | $[1.80, 2.72]$ |
| 5515 | . | 0.43 | $[0.28, 0.66]$ | 3.68 | $[0.79, 31.65]$ | 1.97 | $[1.71, 2.30]$ |
| 5523 | . | 0.56 | $[0.40, 0.85]$ | 3.65 | $[0.78, 36.13]$ | 1.97 | $[1.67, 2.28]$ |
| 5524 | . | 0.45 | $[0.29, 0.67]$ | 3.13 | $[0.63, 29.46]$ | 1.90 | $[1.62, 2.17]$ |
