# Supplementary material for: Models of SIV rebound after treatment interruption that involve multiple reactivation events
Source: PLoS Comput Biol. 2020 Oct 1;16(10):e1008241. doi: 10.1371/journal.pcbi.1008241 (PMC7529301; doi:10.1371/journal.pcbi.1008241)
Supplement: S2 Table — The Watanabe-Akaike information criterion (WAIC) is averaged over 10 MCMC runs to account for Monte-Carlo error, which is indicated by the standard error of the mean (SEM). The variance k^≡Vars[logL(Di|ps)] in Eq 11 can be interpreted as the effective number of parameters. (PDF) [file pcbi.1008241.s009.pdf]

Model	WAIC	(±SEM)	ΔWAIC	(±SEM)	$\widehat{k}$	(±SEM)
multiple-reactivation model (MRM)	346.3	(±0.3)	–		37.8	(±0.2)
conditionally-deterministic MRM	348.4	(±0.4)	2.1	(±0.5)	38.2	(±0.3)
single-reactivation model	357.8	(±0.7)	11.5	(±0.8)	42.3	(±0.4)
